# miRNA Expression Profiling of the Murine TH-*MYCN* Neuroblastoma Model Reveals Similarities with Human Tumors and Identifies Novel Candidate MiRNAs

**DOI:** 10.1371/journal.pone.0028356

**Published:** 2011-12-02

**Authors:** Marta Terrile, Kenneth Bryan, Lynsey Vaughan, Albert Hallsworth, Hannah Webber, Louis Chesler, Raymond L. Stallings

**Affiliations:** 1 Department of Cancer Genetics, Royal College of Surgeons in Ireland, Dublin, Ireland; 2 Ireland and National Children's Research Centre, Our Lady's Children's Hospital, Crumlin, Dublin, Ireland; 3 Division of Cancer Therapeutics, The Institute of Cancer Research, Sutton, Surrey, United Kingdom; Istituto Dermopatico dell'Immacolata, Italy

## Abstract

**Background:**

MicroRNAs are small molecules which regulate gene expression post-transcriptionally and aberrant expression of several miRNAs is associated with neuroblastoma, a childhood cancer arising from precursor cells of the sympathetic nervous system. Amplification of the MYCN transcription factor characterizes the most clinically aggressive subtype of this disease, and although alteration of p53 signaling is not commonly found in primary tumors, deregulation of proteins involved in this pathway frequently arise in recurrent disease after pharmacological treatment. TH-*MYCN* is a well-characterized transgenic model of MYCN-driven neuroblastoma which recapitulates many clinicopathologic features of the human disease. Here, we evaluate the dysregulation of miRNAs in tumors from TH-*MYCN* mice that are either wild-type (TH-*MYCN*) or deficient (TH-*MYCN*/p53ER^TAM^) for the p53 tumor suppressor gene.

**Principal Findings:**

We analyzed the expression of 591 miRNAs in control (adrenal) and neuroblastoma tumor tissues derived from either TH-*MYCN* or TH-*MYCN*/p53ER^TAM^ mice, respectively wild-type or deficient in p53. Comparing miRNA expression in tumor and control samples, we identified 159 differentially expressed miRNAs. Using data previously obtained from human neuroblastoma samples, we performed a comparison of miRNA expression between murine and human tumors to assess the concordance between murine and human expression data. Notably, the miR-17-5p-92 oncogenic polycistronic cluster, which is over-expressed in human *MYCN* amplified tumors, was over-expressed in mouse tumors. Moreover, analyzing miRNAs expression in a mouse model (TH-*MYCN*/p53ER^TAM^) possessing a transgenic p53 allele that drives the expression of an inactive protein, we identified miR-125b-3p and miR-676 as directly or indirectly regulated by the level of functional p53.

**Significance:**

Our study represents the first miRNA profiling of an important mouse model of neuroblastoma. Similarities and differences in miRNAs expression between human and murine neuroblastoma were identified, providing important insight into the efficacy of this mouse model for assessing miRNA involvement in neuroblastoma and their potential effectiveness as therapeutic targets.

## Introduction

Neuroblastoma is among the most common of childhood tumors and accounts for 15% of pediatric cancer deaths. The disease is clinically heterogeneous, with behavior ranging from spontaneous regression to rapid progression. Multiple genetic abnormalities have been identified that are predictive of clinical outcome and that play important roles in neuroblastoma pathogenesis [1]. Amplification of the gene encoding the *MYCN* transcription factor is the most potent genetic predictor of poor patient outcome and delineates a distinct genetic subtype of high-risk neuroblastoma [2]. As a transcription factor, MYCN directly regulates the expression of a large set of genes and microRNAs (miRNA), whose major functions include regulation of cell cycle progression, proliferation, differentiation and apoptosis. 3–5.

MiRNAs are small molecules (22–24 nucleotides) of RNA which negatively regulate gene expression at a post-transcriptional level. The binding of miRNAs to complementary sites on the 3′ UTRs of protein coding mRNA sequences results in either degradation of the mRNA or translational inhibition [6]. MiRNAs are involved with the regulation of many normal physiological processes and their dysregulation contributes to the pathogenesis of virtually all forms of cancer [7], [8], including neuroblastoma [9]–[11]. A number of miRNA expression profiling studies have shown that miRNA expression is deregulated in *MYCN* amplified (MNA) tumors relative to *MYCN* non-amplified tumors (non-MNA), and that miRNA expression signatures are independently predictive of patient survival [12]–[17]. Moreover, functional studies have also demonstrated that specific miRNAs promote neuroblastoma cell proliferation in *in vitro* and *in vivo* assays [18], [19], or alternately, act to suppress tumorigenesis by stimulating/reactivating apoptosis [20], [21] and differentiation [22] or by inhibiting cell invasion [23].

TH-*MYCN* is a murine transgenic model of neuroblastoma that is increasingly used for a variety of molecular and pre-clinical studies [24]. Tumorigenesis is driven by neural crest-specific expression of a human *MYCN* transgene. These animals develop aggressive tumors that replicate major features of MNA high-risk disease [24]–[26]. The oncogenic action of MYC proteins is strongly enhanced in a variety of cancers by loss of p53, a functional interaction that has been replicated by the introduction of MYC-driven transgenic models in a p53-deficient background [27]. Alterations in p53 signaling are rarely observed in neuroblastoma patients at diagnosis. However abnormalities in multiple p53 pathway members emerge after pharmacological treatment, correlating with therapeutic resistance and reinforcing the notion that strong selective pressure for p53 inactivation exists in MYCN-driven neuroblastoma [28], [29]. Indeed, murine neuroblastoma is strongly enhanced by placing TH-*MYCN* into a p53 insufficient background, and this is concomitant with reduced apoptosis and chemotherapy resistance [25]. The interplay between MYCN and p53, however, is complex. MYCN directly up-regulates p53 transcriptional expression [30], but also inhibits p53 through the direct up-regulation of MDM2, which encodes for a ubiquitin ligase that targets tumor protein p53 for proteasomal degradation [31]. Taken together these observations highlight the utility of these preclinical models for studies of MYCN-p53 control mechanisms [25].

Since miRNA expression levels are associated with clinical tumor grade, metastasis and overall survival in neuroblastoma, miRNAs are potential targets for therapeutic treatment. The availability of transgenic models with altered expression of individual oncogenes and tumor suppressor genes in a genetically homogeneous background present an ideal opportunity to test efficacy of novel miRNA mediated therapeutics [32]. However, knowledge about miRNA expression profiles in the TH-*MYCN* transgenic mouse model or its variants is lacking, particularly the degree to which murine miRNA expression profiles parallel those of human neuroblastoma. Here, we generated miRNA profiles of murine tumors and adrenal tissues derived from TH-*MYCN* mice in both wild-type and mutant p53 deficient backgrounds. Our analyses reveal similarities and differences between the miRNA expression profiles of human and mouse neuroblastoma and have allowed us to assess the impact of p53 haploinsuffiency on miRNA expression. By comparing the miRNA profile of murine and human tumors, we demonstrate the extent to which the transgenic mouse model can be used for miRNAs related studies.

## Results

### MiRNA expression profiling of murine tumors

In order to identify miRNAs associated with neuroblastoma tumorigenesis, we analyzed the expression of 591 murine miRNAs in 22 mouse tumors using TaqMan low density arrays. Nine tumors were derived from TH-*MYCN* mice (wild-type for p53) and 13 from TH-*MYCN* mice in a p53 deficient background (TH-*MYCN*/p53ER^TAM^). As a control group, we profiled 12 adrenal glands (three from TH-*MYCN*; five from TH-*MYCN*/p53ER^TAM^ mice, and four from wild-type mice) coming from young mice (7 days after birth). Adrenals coming from TH-*MYCN* and wild-type mice were stained with hematoxylin and eosin, confirming that the tissues were histologically normal in the transgenic mice (Figure S1). After primary profiling, the data set was filtered to include 440 miRNAs with expression in at least 10 of the samples (Figure S2).

**Figure 1:**
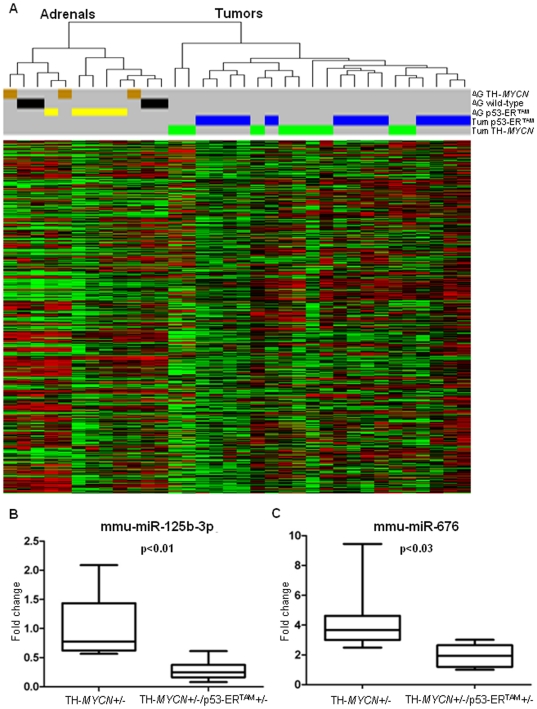
Hierarchical cluster and expression analysis of mouse tumors. (**A**) Hierarchical clustering (n = 440) of mouse samples. Cluster “Adrenals” contains all the adrenals, while all the tumors samples fall into the cluster “Tumors” The overall miRNAs profile did not resolve the TH-*MYCN* (green) and TH-*MYCN*/p53ER^TAM^ (blue) tumors, or the adrenals derived from wild type (black), TH-*MYCN* (brown) and TH-*MYCN*/p53ER^TAM^ (yellow). (**B**)–(**C**). Expression of miR-125b-3p and miR-676 in TH-*MYCN* (n = 9) and TH-*MYCN*/p53ER^TAM^ tumors (n = 13). The miRNAs are significantly down-regulated in the tumors lacking half of the active p53 protein, after Bonferroni multiple comparison correction (respectively p<0.01 and p<0.03).

Unsupervised hierarchical clustering based on the expression of the total dataset of 440 miRNAs (Table S1) resolved tumors from adrenal gland controls (Figure 1A). Before using the adrenals as a unique group of controls regardless their genotype, we analyzed the miRNA expression of TH-*MYCN* (n = 8) and wild type (n = 4) adrenals finding no statistically significant differences. Then we compared all tumors (n = 22) to all adrenal controls (n = 12). Using the Wilcoxon Rank Sum Test corrected for multiple comparisons (n = 440), we found 159 miRNAs differentially regulated (p<0.05) in tumors: 81 were over-expressed (>2-fold) and 78 were under-expressed (>2-fold) (Table S2). Thus, miRNA expression in tumor tissue compared to a developmentally differentiated tissue precursor is significantly differentially regulated. Intriguingly, haploinsufficiency for p53 did not dramatically alter miRNA expression patterns as the tumor samples clustered together regardless their genotype.

Since the miRNA profiles did not show any major differences based on the presence of the p53-ER^TAM^ allele, we measured expression of the endogenous p53 transcript in tumors to confirm haploinsufficiency. The primers were designed to detect expression of the endogenous wild-type p53 allele, and not the mutant p53-ER^TAM^ allele (Figure S3A). Five TH-*MYCN* and 6 TH-*MYCN*/p53ER^TAM^ tumors were analyzed through quantitative PCR. All TH-*MYCN*/p53ER^TAM^ animals showed a reduced level of wild-type p53 mRNA (t-test, p<0.0001) (Figure S3B). These data demonstrate that no compensatory up-regulation of the endogenous p53 allele occurs in TH-*MYCN*/p53ER^TAM^ tumors.

Although cluster analysis based on all miRNAs did not distinguish tumors with respect to p53 genotype, miR-125b-3p and miR-676 were significantly down-regulated (p<0.03) in TH-*MYCN*/p53ER^TAM^ tumors (Figure 1B–C) based on the Wilcoxon Rank Sum Test with p-values corrected for multiple comparisons (n = 440).

### Cross-Comparison of miRNA expression in human and murine tumors

In order to assess the relevance of the mouse model as a representative platform for studies of important miRNA targets modulated in human neuroblastoma, we first compared the set of miRNAs differentially expressed in mouse and human tumors, using previously published miRNA expression profiles of human neuroblastoma [12], [16]. In total, 296 mature miRNAs are conserved between mouse and human in the miRBase version 16 (Table S3). Taking into account only conserved miRNAs (Figure S4A), a total of 63 miRNAs have been identified as differentially expressed in human MNA versus non-MNA tumors in at least one published study [12], [16]. Among the 63 conserved miRNAs differentially expressed between human MNA versus non-MNA tumors, 29 (46%) were differentially expressed between mouse tumors and adrenals (Figure S4B). This amount of overlap between differentially expressed miRNAs in human versus mouse tumors is statistically significant (p<0.01) according to a hypergeometric distribution [33]. The majority of the miRNAs had alterations in expression that were consistent between the two species, except for miR-323-3p, miR-369-5p, miR-410, miR-411, miR-433, miR-494 and miR-130a, which were expressed discordantly in the tumors from the two different species (Table 1). It is interesting to note that 6 out of 7 of the miRNAs that were inconsistent in the mouse/human comparison map to a large cluster on chromosome 14q in humans (mouse chromosome 12). Overall, these results showed high concordance in miRNA expression between murine and human neuroblastoma, with the aforementioned exceptions.

**Table 1:** miRNAs differentially expressed in mouse and human tumors.

MIRNA NAME	MOUSE TUMORS	HUMAN MNA TUMORS	
		Bray et al.	Mestdagh et al.
miR-130b	**Up**	**Up**	
miR-17	**Up**	**Up**	
miR-18a	**Up**	**Up**	**Up**
miR-19a	**Up**	**Up**	**Up**
miR-19b	**Up**	**Up**	
miR-20a	**Up**	**Up**	**Up**
miR-20b	**Up**	**Up**	**Up**
miR-25	**Up**	**Up**	
miR-9	**Up**	**Up**	**Up**
miR-9*	**Up**	**Up**	
miR-93	**Up**	**Up**	
miR-323-3p	**Up**	**Down**	
miR-369-5p	**Up**	**Down**	
miR-410	**Up**	**Down**	
miR-411	**Up**	**Down**	
miR-433	**Up**	**Down**	
miR-494	**Up**	**Down**	
miR-152	**Down**	**Down**	
miR-204	**Down**	**Down**	
miR-26a	**Down**		**Down**
miR-26b	**Down**		**Down**
miR-30a	**Down**		**Down**
miR-30a*	**Down**	**Down**	
miR-30d	**Down**		**Down**
miR-30e	**Down**		**Down**
miR-30e*	**Down**	**Down**	
miR-328	**Down**	**Down**	**Down**
miR-491	**Down**	**Down**	**Down**
miR-130a	**Down**		**Up**

Up = Over-expressed in MNA tumors; Down = under-expressed in MNA tumors.

**Figure 2:**
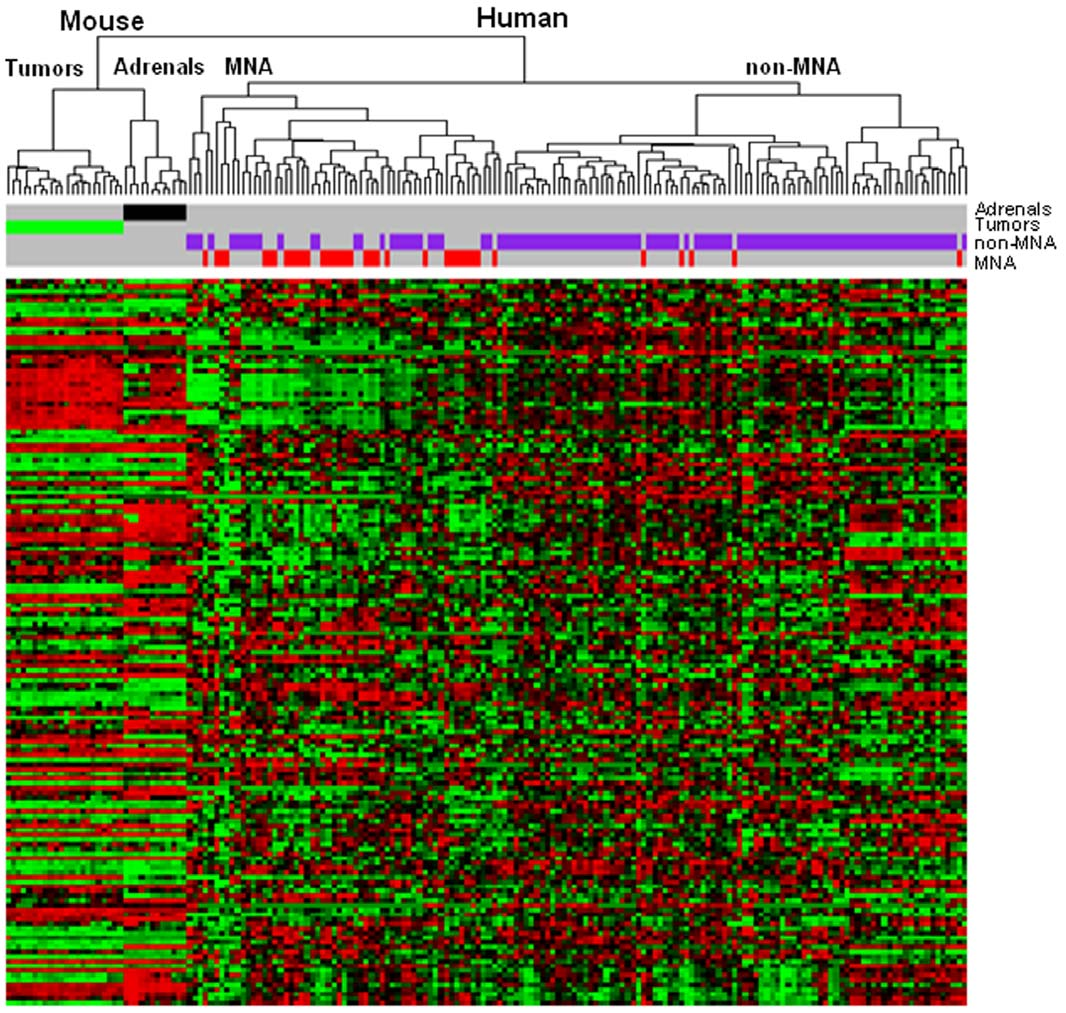
Overlapping of miRNAs deregulated in human and mouse tumors and hierarchical clustering on all miRNAs. Hierarchical clustering based on the overall miRNAs conserved and present in both the studies (n = 155). Cluster “Mouse” contains all the murine samples split in two branches (“Tumors” and controls “Adrenals”). Cluster “Human” includes all the human tumors split (p<0.001) in a “MNA” and a “non-MNA” branch with an enrichment (52.5%) and an under-representation (5.7%) of MNA samples (red), respectively.

Using data previously obtained in our laboratory, we directly compared the miRNA profile of human and mouse tumors. The cohort of 146 human tumors included 36 tumors MNA and 110 samples non-MNA [12]. Comparison between human and mouse tumors was complicated by the fact that not all miRNAs in the current study were included in our prior study and by the non-conservation of many miRNAs between the species. 155 miRNAs were profiled in both murine and human tumors profiled by Bray et al. [12]. Unsupervised hierarchical cluster analysis with this set of 155 miRNAs of murine and human samples (n = 180 samples) indicated that murine adrenals and tumors clustered closer together relative to human tumors (Figure 2). However, hierarchical cluster analysis maintains the distinction between murine tumors and adrenals. Interestingly, this subset of miRNAs was differentially expressed in MNA versus non-MNA human tumors (χ2-test, p<0.001). The MNA branch of the dendrogram contained 31/36 (86%) of the MNA tumors, while the non-MNA branch contained 5/36 (14%) of the MNA tumors.

**Figure 3:**
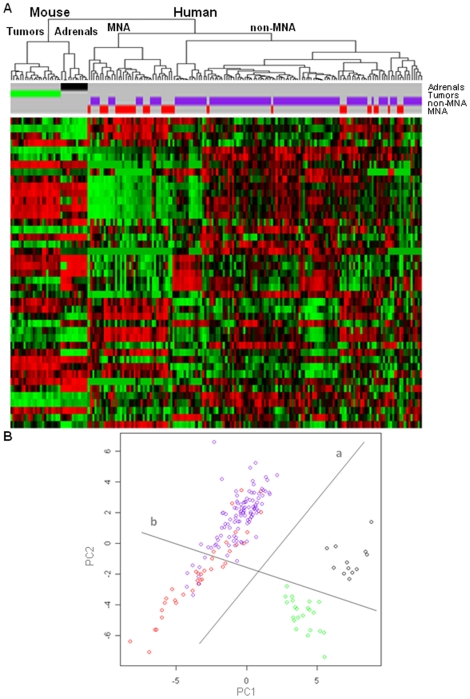
Analysis of human and mouse samples based on miRNAs (n = 43) deregulated in human MNA. (**A**) Hierarchical clustering (n = 43). Cluster “Mouse” contains all the mouse samples, split in two branches “Tumors” and controls “Adrenals”. Cluster “Human” includes all the human samples split in two branches which shows an enrichment (p<0.05) for MNA tumors (59.5%, in cluster “MNA”) and an under-representation of MNA samples (12.9%, in cluster “non-MNA”), respectively. (**B**) Principal component analysis. Line “a” splits the samples based on the species: human (left) and mouse (right). Line “b” shows a distribution of the samples according to MYCN expression: a clear split in the mouse and an enrichment (p<0.001) for human MNA samples (under). Mouse tumors (green), mouse adrenals (black), human MNA (red) and human non-MNA (purple).

Since the mouse model over-expresses human *MYCN*, we hypothesized that there might be similarities with human MNA tumors for MYCN regulated miRNAs. Hierarchical clustering based on the conserved miRNAs differentially expressed between human MNA and non-MNA tumors [12] revealed that mouse tumors more closely resemble adrenals than human MNA tumors, (Figure 3A). As expected, there was a branch of human tumors significantly enriched (p<0.005) for MNA human tumors 22/37 (60%).

Principal component analysis (PCA) was carried out in order to identify miRNAs that could account for the distinction between human and mouse tumors and between human MNA and non-MNA tumors. Figure 3B depicts the two top ranking principal components, PC1 and PC2. The distribution of the samples in PCA discriminates the two species mainly based on PC1, while the PC2 axis highlighted a distribution of the samples according to *MYCN* expression. The human tumors segregated by line b (Figure 3B) were significantly enriched for MNA (25/36, 69%; χ2-test; p<0.001). To identify the miRNAs involved in these events, we analyzed the miRNAs of PC1 and PC2 (Table S4). The miRNAs that contributed most prominently to PC1 (human - mouse split) were miR-93 and miR-19a, with a lesser contribution from miR-19b, miR-20a and miR-130b, while the miRNAs that contributed most significantly to PC2 (MYCN high versus low expression) were miR-17, miR-25, miR-20b and miR-15b. Interestingly, 8 of the top 20 PC2 miRNAs were deregulated in both human and mouse tumors (Table 1). Moreover, 11 of the first 20 miRNAs contributing to each component were common to PC1 and PC2, clarifying how both the shifts, due to species and to human MYCN expression, involved the two axis. These results suggest that human MYCN may regulate similar miRNAs in human and mouse.

**Figure 4:**
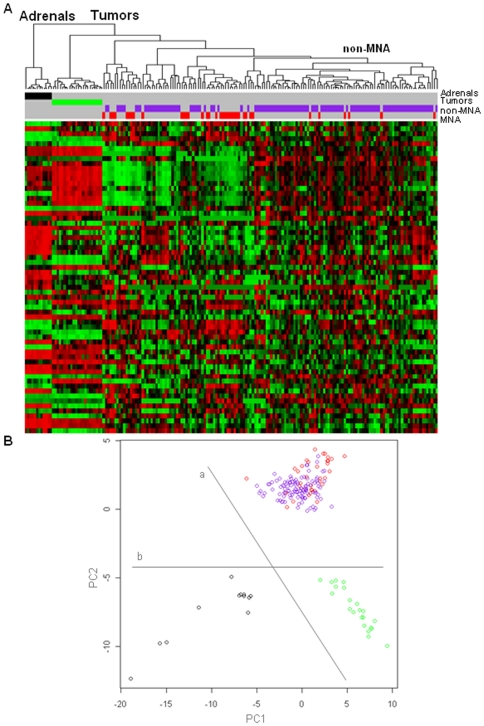
Analysis of human and mouse samples based on miRNAs (n = 63) deregulated in mice tumors. **A.** Hierarchical clustering. Cluster “Adrenals” contains all the mouse adrenals. Cluster “Tumors” collects all the “Mouse” and “Human” tumors, highlighting a branch enriched (p<0.01) for human non-MNA samples (90.7%). **B.** Principal component analysis. Line “a” splits the samples based on the species: mouse (under) and human (above). Line “b” separates the samples according to the presence of the pathology: controls (left) and tumors (right). Mouse tumors (green), mouse adrenals (black), human MNA (red) and human non-MNA (purple).

We finally restricted our analysis to conserved miRNAs with differential expression between murine tumors and adrenals. Interestingly, the hierarchical clustering based on this subset of miRNAs (n = 63) distinguished tumors and adrenals independent of species. Unlike previous analyses, these miRNAs delineated murine tumor tissue from adrenal with higher specificity (Figure 4A). The analysis did not clearly distinguish human MNA and non-MNA tumors, however it identified a group of samples enriched (p<0.01) for non-MNA 68/75 (91%) allowing the mouse tumors to cluster closer to the human MNA samples.

PCA again revealed an interspecies difference (PC2 axis), while the PC1 axis discriminated tumors and controls (Figure 4B). To identify the miRNAs underlying these differences, we considered the loadings of the two principal components (Table S5). The most significant contribution to PC1 was from miR-10a, miR-30a, miR-30d, let-7f, miR-22 and miR-23b, ranked in order. Consistent with the interspecies shift based on PC2, 3 out of the first 5 implicated miRNAs (miR-411, miR-410, miR-382, miR-495 and miR-494) were differentially modulated in human and mouse samples (Table 1).

## Discussion

The importance of highly representative murine models for studying human pathologies and for developing new pharmacological treatments is established [32], however it is necessary to determine on a case-by-case basis the degree to which each model faithfully mimics human pathology. The TH-*MYCN* murine model for neuroblastoma has been well-characterized from the histopathological, gene expression and genome instability point of view [24]–[26], [34]. Here, we identify miRNAs that are differentially expressed in this model (relative to adrenal controls). Cross-comparison between this and previous human studies reveals similarities and differences between the mouse model and the human pathology with regard to miRNA expression. The miRNA profile across murine tumors is more homogeneous than that observed in human tumors, perhaps a reflection of the inbred genetic tumor environment in murine versus human tumors. This supports the use of mouse models for studying pathological events in a more defined genetic background, with reduced heterogeneity due to genetic variation that often affects human studies.

In total, 22 conserved miRNAs were differentially expressed across both murine tumor versus adrenal and human MNA versus non-MNA tumors (as identified in at least one of two independent studies on human tumors [12], [16]). This degree of similarity between human and mouse tumors is consistent with interspecies comparisons for other types of tumors [35]. Notably, several members of the miR-17-92 polycistronic cluster, which are highly up-regulated in human MNA tumors [36], were up-regulated in mouse tumors relative to the adrenal glands. This polycistronic cluster has demonstrated oncogenic functions in neuroblastoma through targeting multiple genes of the TGFB signaling pathway [18], [19]. Similarly, miR-9, which is positively regulated by MYCN in human neuroblastoma [37], was also significantly up-regulated in the mouse tumors. In addition, miR-152 is down regulated in both mouse and human neuroblastoma, having a documented tumor suppressor function in human neuroblastoma [38]. Overall, our results in the mouse model indicate that the over-expression of MYCN strongly deregulates miRNAs expression, similar to the situation in human neuroblastoma.

The major tumor suppressor miRNA, miR-34a, which is down-regulated in human neuroblastoma, was not changed in the mouse tumors and represents an example of where the mouse model is inconsistent with human neuroblastoma. Many factors are likely to account for the differences in miRNA expression between human and mouse neuroblastoma. For example, in addition to MYCN, large-scale genomic imbalances have been demonstrated to influence miRNA expression in human tumors [12]. Although some similarities in genomic imbalances have been demonstrated between murine and human tumors [34], there are also extensive differences between human and mouse genomes resulting from the accumulation of approximately 200 chromosomal rearrangements since the evolutionary divergence of rodent and primate lineages. It is also important to realize that neuroblastoma originate from fetal neuroblasts [39], so that some differences in miRNA expression between tumors and adrenal glands (from 7 day old mice) could relate to differentiation associated changes rather than cancer specific alterations. This possibility requires further testing by profiling micro-dissected fetal neuroblasts, a technically challenging endeavor.

P53 is a critical tumor suppressor gene inactivated in a variety of cancers. In neuroblastoma, p53 is rarely inactivated in primary tumors, but is often mutated in recurrent tumors. Although the p53 transcription factor is known to regulate a number of miRNAs, including miR-34a, miR-23b, miR-26, miR-30, miR-107 and miR-192 [40]–[45], there was very little difference in miRNA expression patterns between tumors derived from p53 wild-type TH-*MYCN* versus p53 mutant TH-*MYCN* mice, despite significant differences in tumor phenotype when MYCN drives neuroblastoma in a p53 deficient background [25]. This implies that p53 mediated regulation of miRNAs requires complete abrogation of p53 activity rather than p53 haploinsufficiency. Certainly this was the case for p53 regulation of miR-34a, where the effects of p53 were investigated in a p53- homozygous mouse knockout model [44], [46]. Thus, we hypothesize that the modulation of miRNAs induced by p53 is not dose dependent in our model and that the vast majority of miRNAs are not significantly involved in the more aggressive phenotype driven by p53 haploinsufficiency. However, confirmation of this supposition would require the analysis of miRNA expression derived from mice homozygous for mutant p53.

The only two miRNAs that were down-regulated in TH-*MYCN* tumors from TH-*MYCN*/p53ER^TAM^ mice were miR-125b-3p and miR-676. No specific role has thus far been ascribed to miR-676 in human neuroblastoma, which has a seed region that is not identical between the two species. Interestingly, miR-125b has been identified as both a target of p53 [47] and a regulator of the p53 transcript itself [48]. miR-125b inhibits neuroblastoma cell proliferation and promotes cell differentiation in *in vitro* models [49], [50], so that down-regulation of this miRNA could potentially be contributing towards the more aggressive phenotype of the TH-*MYCN*/p53-ER^TAM^ tumor phenotype. This is an interesting possibility requiring further studies.

Although many of the differentially expressed miRNAs were previously identified as being associated with human neuroblastoma (Table 1), this study identifies additional miRNAs not previously correlated with this pathology, indicating a potential role in neuroblastoma pathogenesis. Indeed, many of the most significantly differentiated miRNAs in mouse tumors versus adrenal tissue have never been associated with any form of cancer to date (Table S2). Functional studies are required to corroborate any role that these miRNAs might have in tumorigenesis. Lastly we demonstrate that, despite some differences among the two species, the mouse model recapitulates a pattern of miRNA expression that is dependent on MYCN expression and which is similar to the one observed in human MNA tumors. Our results further support the involvement of miRNAs in neuroblastoma, validating the utility of TH-*MYCN* mouse model for studying the role of miRNAs in this pathology and for possible pre-clinical trials based on miRNA agonist/antagonist molecules.

## Materials and Methods

### Transgenic mice and samples collection

TH-*MYCN* mice were maintained in hemizygotic matings [26]. TH-*MYCN*/p53ER^TAM^ animals were obtained serially crossing TH-MYCN+/− and p53-ER +/− mice [25]. Adrenals were obtained from mice 7 day post birth, while tumors were collected after the onset of the tumor pathology (>40 days post birth). All tissue samples were either frozen in liquid nitrogen or submerged in RNAlater RNA Stabilization Reagent (Qiagen, Crawley, UK) and stored at −80°C. All animals were handled in accordance with institutional guidelines for safe and ethical treatment of mice. The protocol was approved by the U.K. Home Office (License number PPL70/6882).

### RNA isolation

Total RNA extraction was performed using RNeasy (Qiagen), miRNeasy (Qiagen), or mirVana™ miRNA Isolation Kit (Ambion, Austin, Texas) following the manufacturer guidelines.

### Reverse transcription

For messanger RNA (mRNA) cDNA synthesis, total RNA was reverse transcribed using random primers and a TaqMan Reverse Transcription kit (Applied Byosistems, Foster City, CA). Up to 762 rodent mature miRNAs were converted to cDNA (381 miRNAs processed simultaneously per reaction) using the Megaplex™ RT Rodent Pool (Applied Biosystems).

### Quantitative PCR for gene expression

The level of endogenous p53 was analyzed using primers specifically designed for targeting exon 10 and 11 of the transcript (sequences of primers available on request) and SYBR® Green PCR Master Mix (Applied Biosystem). mRNA quantifications were normalized to the housekeeping gene β-actin. Relative quantification of genes expression was determined using the comparative cycle threshold method (2−ΔΔCT).

### Pre-amplification and quantitative PCR of miRNAs

Megaplex retro transcription product was pre-amplified using TaqMan PreAmp Master Mix and Megaplex™ PreAmp Primers, Rodent Pool (Applied Biosystems). The miRNA profile of each cDNA sample was obtained using 384-well microfluidic cards (TaqMan® MicroRNA Array v2.0, Applied Biosystems). As instrument and liquid handling variations were shown to be minimal, no PCR replicates were measured. All quantitative PCR (qPCR) were carried out on the 7900 HT Fast Real-time System (Applied Biosystems).

### Data analysis

Since the presence of a single molecule of target lead to a Ct value of 35, all miRNAs with Ct values greater than 35 were considered not expressed. The mean Ct of a sample was subtracted from the Ct value of each miRNA among that sample (mean centered normalization) before calculating relative expression values [51]. Normalized relative expression (NRQ) of miRNA was calculated with reference to the Ctmax (maximum Ct value for an individual miRNA across all samples) using: NRQ = 2(Ctmax−Ct).

### Significance testing

The statistical significance of miRNA differential expression over sample classes was evaluated by assigning p-values based on the non-parametric Wilcoxon Rank Sum Test. P-values were corrected for multiple comparisons using the Bonferroni method. The statistical significance of the enrichment for a specific genotype among the groups generated by the hierarchical clustering was evaluated using the chi-squared test (χ2-test) on the frequency of observed samples compared to the expected random distribution. The statistical significance of the overlap between the lists of miRNAs was evaluated using the hypergeometric distribution [33].

### miRNA homology identification

An in-house developed Java (v6.0) software was used for comparing the sequences (retrieved from miRBase v16) of human and mouse mature miRNAs. We considered as homologue only miRNAs whose mature transcript showed perfect identity in both length and nucleotide sequence among the two species.

### Cluster analysis and visualization

Hierarchical clustering, heatmap generation, principal component analysis (PCA) and the loadings of PCA were performed using “hclust” (agglomeration method complete based on Spearman rank correlation coefficient), “heatmap.2”, “prcomp” and “pcs$rotation” functions of the R statistical computing language v2.13.0.

## Supporting Information

Figure S1 **Comparison of hematoxylin and eosin stained adrenal glands from wild-type and transgenic mice at day 7 after birth.** The gross size is similar in transgenic versus wild-type mice and there is no evidence of changes in cell morphology indicative of hyperplasia or the development of early proliferative intra-adrenal preneoplastic foci.
(TIF)

Figure S2 **Overview of the data flow and analyses.** Schematic representation of the concept of the project and of the steps in the analysis.
(TIF)

Figure S3 **Analysi-es of p53 endogenous expression in wild type and deficient p53 tumors.** **A.** Schematic representation of the primer design for the specific detection of the endogenous p53 transcript. **B.** Expression of endogenous p53 in TH-*MYCN* and TH-*MYCN*/p53ER^TAM^ tumors. The samples from p53 wild type mice (n = 5) express approximately 2-fold more endogenous p53 (p<0.001) compared to TH-*MYCN*/p53ER^TAM^ tumors (n = 6).
(TIF)

Figure S4 **Comparison between miRNAs differentially expressed in human and mouse neuroblastoma tumors.** **A.** Schematic representation of the clearing process for comparing the lists becoming from studies on different species. **B.** Venn diagram representing the overlapping of miRNAs deregulated in human and mice neuroblastoma. miRNAs differentially expressed in mouse tumors compared to adrenals (pink circle), miRNAs deregulated in human MNA compared to non-MNA in Bray (purple circle) and miRNAs differentially expressed in human MNA compared to non-MNA Mestdagh (green circle).
(TIF)

Table S1 **Mouse miRNAs expressed in greater than 10 samples.**
(XLSX)

Table S2 **miRNAs differentially expressed in mouse tumors compared to adrenals.**
(XLSX)

Table S3 **miRNAs homologue between mouse and human.**
(XLSX)

Table S4 **PCA loadings based on miRNA differentially expressed in human MNA compared to non-MNA.**
(XLSX)

Table S5 **PCA loadings based on miRNA differentially expressed in mouse tumors compared to adrenals.**
(XLSX)
