# Supplementary figures and images for: miRNA Expression Profiling of the Murine TH-MYCN Neuroblastoma Model Reveals Similarities with Human Tumors and Identifies Novel Candidate MiRNAs
Source: PLoS One. 2011 Dec 2;6(12):e28356. doi: 10.1371/journal.pone.0028356 (PMC3229581; doi:10.1371/journal.pone.0028356)

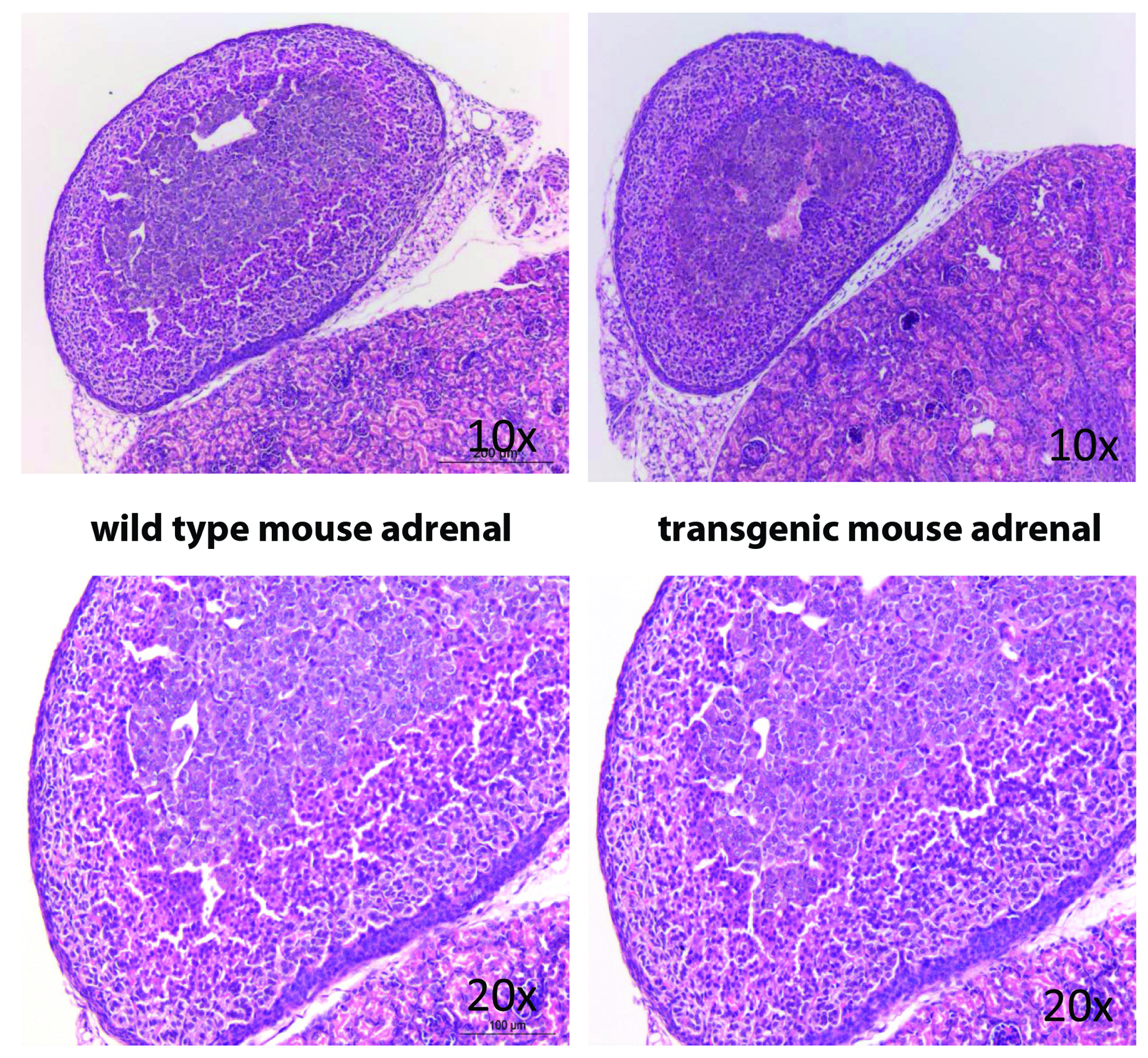

Supplement: Figure S1 — Comparison of hematoxylin and eosin stained adrenal glands from wild-type and transgenic mice at day 7 after birth. The gross size is similar in transgenic versus wild-type mice and there is no evidence of changes in cell morphology indicative of hyperplasia or the development of early proliferative intra-adrenal preneoplastic foci. (TIF) [file pone.0028356.s001.tif]
